# Parenting Style Frequency and Their Sociodemographic Determinants in Buraidah City, Qassim, Saudi Arabia

**DOI:** 10.7759/cureus.41388

**Published:** 2023-07-05

**Authors:** Shaikah N Almudhee, Abdullah M Al Saigul, Amel Sulaiman

**Affiliations:** 1 Family Medicine, Family Medicine Academy, Qassim Health Cluster, Buraidah, SAU; 2 Family Medicine, Ministry of Health, Buraidah, SAU

**Keywords:** saudi arabia, qassim, socioeconomic status, authoritative style, parenting styles

## Abstract

Background

Three parenting styles still form the foundation for today’s research into childhood development. The natural mode of parenting falls somewhere among Baumrind’s parenting styles (authoritative, authoritarian, and permissive). Due to the lack of research that integrates the relationship between parenting styles and socioeconomic status in Saudi Arabia, we decided to study different types of parenting styles and their relationship with sociodemographic status.

Objective

This study aimed to estimate the frequency of parenting styles among adolescent children’s parents in Buraidah City, Qassim, during the year 2021.

Methods

A descriptive cross-sectional community-based study was conducted among 496 parents. A structured questionnaire was used to collect data. The Parenting Style Dimension Questionnaire (PSDQ) was used to identify the parenting style of each participant. Data were analyzed using the Epi Info software version 7.2.5 (Centers for Disease Control and Prevention, Atlanta, GA, USA).

Results

Among the surveyed parents, there were 250 (50.4%) males, and the mean age was 36.7 (±10.6) years. The majority (390, 78.6%) live in a nuclear family with a family size ranging from four to six members (285, 57.5%). A total of 451 (90.9%) parents were educated in secondary school and above. In this study, the common parenting style was the authoritative style (380, 76.6%). The two sociodemographic factors found to be statistically correlated with authoritative style were big family size (P=0.014) and the husband’s income (P=0.012).

Conclusion

The study revealed that the authoritative parenting style was the dominant type among younger parents. However, no factors affect the parents’ style except family size and the husband’s income. It is important to develop parenting education programs to provide parents with the necessary skills and abilities to deal with their children.

## Introduction

According to the definition given by the dictionary, parenting style is “a constellation of parents’ attitudes and behaviors toward children and an emotional climate in which the parents’ behaviors are expressed” [1]. Parenting is universally important in shaping child and adolescent well-being. Research consistently shows that parenting practices and styles are linked to the behavioral and emotional development of adolescents [2]. The importance of parenting arises from its role as a buffer against poverty and delinquent influences, and a mediator of damage [3].

Diana Baumrind divided parenting styles into three typologies: authoritative, authoritarian, and permissive. These were categorized based on two dimensions of parenting behavior and style: (1) demandingness (parent control and demand) and (2) responsiveness (parent acceptance of developmental needs) [4].

Authoritative parents have high expectations for achievements, but they are also warm and responsive. Authoritarian parents have a high level of parental control and only allow one-way communication. These parents often imply harsh punishment as a way to control children’s behavior, and they are not responsive to their children’s needs. On the contrary, permissive parents set very few rules and boundaries. These parents are warm and have high engagement with their children’s needs [5,6]. Socioeconomic status is a multifactorial variable that plays a primary role in learning and developing a foundation for child well-being and positive lifelong behaviors [7,8]. Socioeconomic status directly and indirectly influences the young child’s cognitive, language, social, physical, and emotional development [9].

Diana Baumrind identified those three main styles in the 1960s. Later on, a fourth type of parenting style, “neglectful style,” was added in the 1980s by Eleanor Maccoby and John Martin [10].

In this study, we focused only on Diana Baumrind’s three main styles (authoritative, authoritarian, and permissive); the neglectful parenting style will not be discussed [4].

Our main call in this study was to estimate each parenting style frequency among adolescent children’s parents as per Diana Baumrind’s classification [4] and determine the relationship between the sociodemographic characteristics of the participants and parenting styles.

## Materials and methods

Study design, setting, and population

This was a descriptive cross-sectional facility-based study conducted among adolescents’ parents in Buraidah City, Qassim, Saudi Arabia. The Saudi parents who participated in this study were selected randomly from 40 primary healthcare centers (PHCCs) in Buraidah City. Out of 570 distributed forms, 549 responded. Fifty-three forms were excluded as they did not meet our inclusion criteria, which were Saudi parents, living in Buraidah City, and voluntary participation. Four hundred ninety-six were suitable for analysis, giving a response rate of 90.3%.

Sampling and data collection procedure

Data was collected using a pre-tested questionnaire consisting of two parts. The first part addressed the study participants’ demographic and family socioeconomic status. The second part of the questionnaire measured the use of the participants’ parenting styles according to the Parenting Style Dimension Questionnaire (PSDQ) [11-13] based on Baumrind’s parent typology: authoritative, authoritarian, and permissive [14]. Parents were asked to rank the occurrence of each statement from 1 to 5 as follows: 1 = never, 2 = once in a while, 3 = about half the time, 4 = very often, and 5 = always. The rank from 1 to 5 displayed how often parents exhibited the behaviors mentioned in each question. As we could not retrieve the original forms, nor an Arabic translation, the questionnaire was retrieved from an open source [13] and translated by the principal investigator. A copy of the used questionnaire is presented in the Appendices section.

The principal investigator and data collectors collected the data from parents who were voluntarily willing to participate in this study. They were directed to help participants with low literacy levels to complete the questionnaire and supervise independent participants. Data collectors waited for eligible attendees with the receptionist. They frequently alternated between male and female sections to collect the study sample equally. They conveniently approached adult attendees, inquired whether they have adolescent children, and invited them to voluntarily participate in the study by completing the study questionnaire. Participants were given a hard copy or a direct link to the questionnaire as per their preference. Data collectors supported study participants as per their needs. Participants completed the questionnaire with simple or partial supervision or had to rely on data collectors if they faced major difficulties.

Data analysis

Data were analyzed using the Epi Info software version 7.2.5 (Centers for Disease Control and Prevention, Atlanta, GA, USA). Frequency tables and percentages were presented for categorical variables, including parenting style. Mean and standard deviation or median and quartile range were used for numerical variables and the final score.

The overall score of the Parenting Style Dimension Questionnaire (PSDQ) was used to identify the parenting style of each participant. Parents were categorized into one of the three parenting styles. We collected the overall mean score for each parenting style category and took the highest score as it determined the dominant parenting style of the parent. To identify the dominant style while having a different number of items for each style, we divided the total score for each item by its number of items. Authoritative and authoritarian style sores were divided by 13, while the permissive style score was divided by 4. For each participant, different parenting styles composed a total of 100. The largest segment determines the dominant style for every given participant.

Parenting style proportions were plotted in a pie chart expressing their frequency as the dominant style. The chi-square test was used to compare final scores for categorical variables, while Mann-Whitney or Kruskal-Wallis tests were used to compare medians, as appropriate.

Ethical considerations

The study proposal was reviewed and approved by Qassim Regional Bioethics Committee (approval number: 1442-2185738). Permission for data collection was also taken from Primary Health Care Director Buraidah. Informed consent was obtained from all the participants. The confidentiality of the participants was ensured at all stages of the research.

## Results

Out of 496 respondents, there were 250 (50.4%) males, and the mean age of the participants was 36.7 (+10.6) years. Most of them (454, 91.5%) were currently married. The majority of Saudi parents had secondary and high school education (257, 51.81%). The median number of participants’ children was two. One-quarter of parents have only one child, and another quarter of parents have more than three children. Around half of the participants were living in apartments (240, 48.4%), followed by villas (158, 31.9%), and the majority of the families (343, 69.2%) owned houses. The median number of household inhabitants was five, ranging from two to 14 members. Nuclear family was the most frequently reported type of family (390, 78.6%). However, 46 (8.3%) were extended families.

The majority of our participants and their partners were currently working (360 (72.6%) and 314 (63.3%), respectively). The husband’s income was the main source of family income (358, 72.2%); however, in around one-quarter of families, the wife does participate in family earnings. Around one-quarter of husbands had a monthly income of 7,000 Saudi Arabian Riyals (SAR) or less, and only 66 (13.3%) had more than 15,000 SAR, while around half of wives had no income at all. Only 69 (13.9%) of our participants had other income resources, mostly from private businesses (Table 1).

**Table 1:** Participants’ characteristics (n=496) SAR: Saudi Arabian Riyal

Item	Number	%
Gender		
Male	250	50.4
Female	246	49.6
Participants’ age group (years)		
<30	137	27.8
30-<50	293	59.4
≥50	63	12.9
Partner’s age of participants (years)		
<30	95	19.6
30-<50	337	69.3
≥50	54	11.1
Marital status		
Married	454	91.5
Separated	11	2.2
Divorced	22	4.4
Widowed	9	1.8
Children number		
1-2	367	76.3
3-5	129	26
Housing		
Traditional house	65	12.9
Villa	158	31.9
Apartment	240	48.4
Farmhouse	34	6.9
Ownership		
Owned	343	69.2
Rented	106	21.4
Rent ending with ownership	47	9.5
Currently family members		
0-3 members	57	11.5
4-6 members	285	57.5
>6 members	154	31.1
Family type		
Nuclear family	390	78.6
Single-parent family	60	12.1
Extended family	41	8.3
Stepfamily	5	1
Education		
Primary school or less	45	9.07
Secondary/high school and diploma	257	51.81
College graduate or more	194	39.1
Current job		
Currently working	360	72.6
Disabled or retired and not looking for work	25	5
Unemployed	111	22.4
Partner’s job		
Currently working	314	63.3
Disabled or retired and not looking for work	18	3.6
Unemployed	164	33.06
Family support		
Husband	358	72.2
Husband and wife	130	26.2
Others	8	1.6
Husband’s income (SAR)		
None	22	4.4
3,000-7,000	112	22.6
7,001-10,000	183	36.9
10,001-15,000	113	22.9
≥15,001	66	13.3
Wife’s income (SAR)		
None	260	52.4
3,000-7,000	110	22.2
7,001-10,000	71	14.3
10,001-15,000	29	5.9
≥15,001	26	5.2
Other resources of income		
Yes	69	13.9
No	427	86.1
Income resource type		
Private business	42	60
External support	28	40

**Figure 1:**
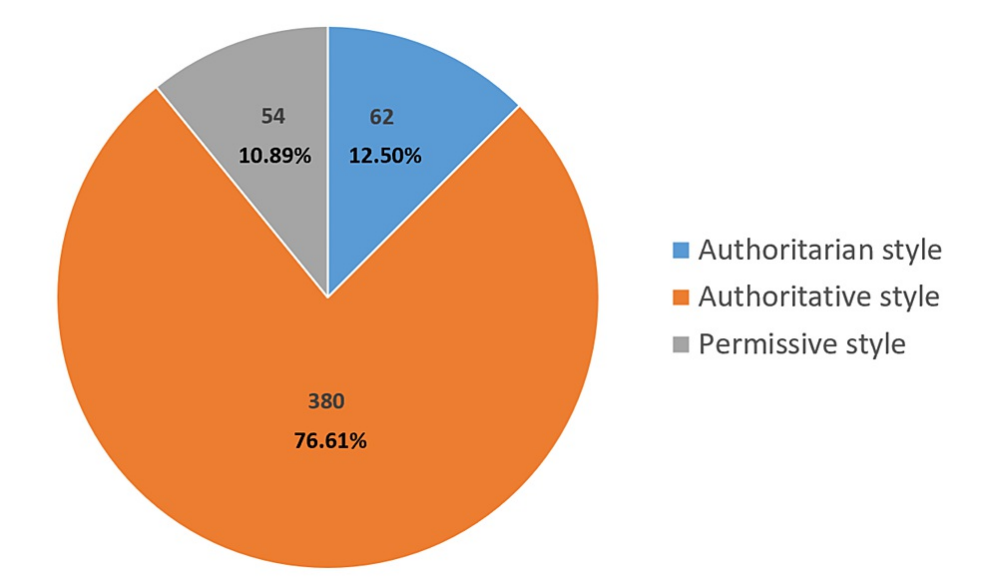
Frequency of parenting styles based on Baumrind’s classification (n=496)

The most common parenting style used among the study population was authoritative (380, 76.6%), followed by authoritarian (62, 12.5%), while only 54 (10.9%) used the permissive style (Figure 1).

Table 2 shows the comparison of each style frequency with respect to the sociodemographic characteristics of the participants. There was no statistical difference in parenting style with respect to gender, education level, occupation, or family type. There were no parenting style differences based on income contribution from spouses (P=0.23). However, the husband’s income was significantly associated with parenting style. The husband’s income of more than 10,000 SAR was associated with authoritative style (P=0.012). Those who lived in traditional houses had less authoritative style frequency than those who lived in other housing types (59.4% and 79.2%) (P=0.002).

**Table 2:** Association between participants’ sociodemographic characteristics and parenting styles (n=496) *Statistically significant SAR: Saudi Arabian Riyal

Item	Authoritarian	Authoritative	Permissive	P-value
Gender				
Male	28 (11.2%)	199 (79.6%)	23 (9.2%)	0.27
Female	34 (13.8%)	181 (73.6%)	31 (12.6%)	
Housing				
Traditional houses	13 (20.3%)	38 (59.4%)	13 (20.3%)	0.002
Non-traditional houses	49 (11.3%)	342 (79.2%)	41 (9.5%)	
Family support				
Husband	49 (13.69%)	268 (74.86%)	179 (11.45%)	0.23
Husband and wife	11 (8.46%)	106 (81.54%)	13 (10%)	
Husband’s income				
10,000 SAR or less	41 (12.9%)	232 (73.2%)	44 (13.9%)	0.012*
10,001 or more	21 (11.7%)	148 (82.7%)	10 (5.6%)	
Wife’s income				
10,000 SAR or less	56 (12.7%)	338 (76.6%)	47 (10.7%)	0.85
10,001 or more	6 (10.9%)	42 (76.4%)	7 (12.7%)	
Current job				
Currently working	41 (11.4%)	281 (78.1%)	38 (10.6%)	0.41
Not working	21 (15.4%)	99 (72.8%)	16 (11.8%)	
Education				
Secondary school or less	38 (12.6%)	231 (76.5%)	33 (10.9%)	0.99
College and higher education	24 (12.4%)	149 (76.8%)	21 (10.8%)	
Family type				
Nuclear family	55 (14.1%)	291 (74.6%)	44 (11.3%)	0.08
Others	7 (6.6%)	89 (83.96%)	10 (9.4%)	
Mean age	34.5 (±10.9)	37.5 (±10.2)	33.7(±12.0)	0.01*
Median household members	4	5	4	0.014*

There was a small but significant difference between the mean age of each dominant parenting style: authoritarian, 34.5 (±10.9); authoritative, 37.5 (±10.2); and permissive, 33.7 (±12.0) (P=0.01). At the same time, there was no difference in the median number of participants’ children for each parenting style as each group had a median of two (P=0.388). Similarly, the median for household members was quite similar, 4, 5, and 4, for authoritarian, authoritative, and permissive, respectively, and the difference was statistically significant (P=0.014) (Table 2).

## Discussion

In this study, the authoritative parenting style was found to be the most commonly used style among participating families. This finding matched four previous studies that described a similar observation [11,14-16].On the contrary, one of the previous studies done in a junior high school in Surabaya in 2018 found that authoritarian was the most widely applied parenting style according to the perception of adolescents (49.9%). Permissive was the least applied parenting style (12.2%) [17]. Another study done in the USA revealed that the permissive style was the dominant parenting style [18].

In our study, the percentage of male gender regarding authoritative parenting style was slightly higher than females; this could be due to the Eastern community culture as the father usually is the head of the family [18]. Inconsistently, in another study conducted on Middle Eastern parents by Dwairy et al. [14], the percentage of females who had an authoritative parenting style was higher than males.

Moreover, the study revealed that the authoritarian parenting style came in second rank among other parenting types, and the percentage of female parents who used the authoritarian style was slightly higher than males. These findings were inconsistent with the results from two previous studies conducted in the USA and Middle East countries, in which the percentage of female parents was lower than males [14,18]. Similarly, our study found that the least used style was the permissive parenting style as in previous studies [1,11,15]. Female parents used this style more than male parents. Additionally, a study done in 2019 among parents living in the Western suburbs of Limassol in Cyprus found that an authoritarian parenting style was more common in fathers than in mothers. Also, older parents were more authoritative than younger parents. Younger parents have a more permissive parenting style [19].

The objective of this study was to test the influence of sociodemographic characteristics of the study participants on their parenting styles. We did not find any statistical association between gender type, education level, occupation, or family type and parenting style. However, a significant statistical relationship was found between the effect of the husband’s income and authoritative parenting style; this finding was inconsistent with the results of the study by Dwairy et al. [14], where the economic level was positively correlated with the permissive style. Another study conducted by Hadjicharalambous and Demetriou [19] found that mothers with low income were more authoritarian than fathers with low income.

There are some limitations that should be considered while interpreting the findings of this study. We cannot generalize the results of the study to other communities since the study population was mostly from middle or high social classes and was selected from one city only. Convenience sampling used in the methodology disrupted the representation of study participants to the general population. The collection of data using a self-administrated questionnaire may affect the quality of the data obtained from illiterate parents compared to the quality of data from educated parents. However, the data collectors explained the questions to illiterate parents to minimize this bias. There is a possibility of social desirability bias. However, we assume it to be of less importance as the data was collected without personal identification and judgment.

## Conclusions

This study highlighted the parenting styles used by parents in the Qassim region of Saudi Arabia. The authoritative parenting style was the dominant type among younger parents. Statistically, no factors affected the parents’ style except family size and husbands’ income, which shows a significant statistical correlation. This can be due to the local culture where fathers were usually supporting and heading their families. It is important to develop a policy and parenting education program to provide the necessary skills and abilities for parents to deal with their children.

## Appendices

Questionnaire

Section 1: Personal Data and Other General Information

Gender: Male (  )      Female (  )      

Age:

Age of husband/wife:

Nationality:    Saudi (  )            Non-Saudi (  )

Marital status:       Married (  )     Separated (  )       Divorced (  )        Widowed (  )

Do you have children in the age group of 10-19 years? Yes (  )          No (  )        

If yes, how many? ( )

Section 2: Family Socioeconomic Status

Housing:

Type of housing:

(  ) Traditional house

(  )  Villa

(  )  Appartement

(  )  Farmhouse

Ownership:

(  )  Owned

(  )   Rented

(  )   Rent ending with ownership

How many people are currently living in your household, including yourself? ( )

Type of family:

(  )  Nuclear family (couple with their own children)

(  )  Single-parent family (single parent with his/her children)

(  )  Extended family (couples and their children and grandparents)

(  )  Stepfamily (with one or both parents having children from another marriage)

Education:

What is the highest level of education you have completed?

(  )   Uneducated

(  )   Can read and write

(  )   Primary school

(  )   Secondary school

(  )   High school

(  )   Diploma/AA degree/technical school training

(  )   College graduate (BA or BS)

(  )   Graduate school degree: Master’s or Doctorate degree (MD, PhD, or JD)

Employment:

What is your current work situation?

(  )   Working full time

(  )   Working part-time

(  )   Unemployed and not looking for work

(  )   Unemployed and looking for work

(  )   Disabled or retired and not looking for work

(  )   Currently in school

Partner’s current work situation:

(  )  Working full time

(  )  Working part-time

(  )  Unemployed and not looking for work

(  )  Unemployed and looking for work

(  )  Disabled or retired and not looking for work

(  )  Currently in school

Who earns income to support your family?

(  )  Husband 

(  )  Wife

(  )  Both the husband and wife

(  )  Others (Please specify:     )

What is the estimated total steady husband income?

(  )  None

(  )  Less or equal to 3,000 SAR

(  )  From 3,001 to less or equal to 6,999 SAR

(  )  From 7,000 to less or equal to 13,999 SAR

(  )  From 14,000 to less or equal to 29,999 SAR

(  )  More or equal to 30,000 SAR

What is the estimated total steady wife income?

(  )  None

(  )  Less or equal to 3,000 SAR

(  )  From 3,001 to less or equal to 6,999 SAR

(  )  From 7,000 to less or equal to 13,999 SAR

(  )  From 14,000 to less or equal to 29,999 SAR

(  )  More or equal to 30,000 SAR

Do you have other resources to support your family?

(  )  Yes

(  )  No

If you answered “Yes” to the above question, what resources do you use?

(  )  Private business

(  )  Citizen’s account program/Taqat or hafez

(  )  Social security system

(  )  Public pension agency/general organization for social insurance

Please estimate the range of monthly income of the previous question.

(  )  Less or equal to 300 SAR

(  )  From 301 to less or equal to 699 SAR

(  )  From 700 to less or equal to 999 SAR

(  )  From 1,000 to less or equal to 2,999 SAR

(  )  From 3,000 to less or equal to 6,999 SAR

(  )  From 7,000 to less or equal to 13,999 SAR

(  )  From 14,000 to less or equal to 29,999 SAR

(  ) More or equal to 30,000 SAR

Section 3: Parenting Style (Source: Parenting Styles and Dimensions Questionnaire [13])

Authoritative parenting style:

1. I am responsive to my child’s feelings and needs.

Rate your response. Choose from 1 (never) to 6 (always):

1 - 2 - 3 - 4 - 5 - 6

2. I take my child’s wishes into consideration before I ask him/her to do something.

Rate your response. Choose from 1 (never) to 6 (always):

1 - 2 - 3 - 4 - 5 - 6

3. I explain to my child how I feel about his/her good/bad behavior.

Rate your response. Choose from 1 (never) to 6 (always):

1 - 2 - 3 - 4 - 5 - 6

4. I encourage my child to talk about his/her feelings and problems.

Rate your response. Choose from 1 (never) to 6 (always):

1 - 2 - 3 - 4 - 5 - 6

5. I encourage my child to freely “speak his/her mind,” even if he/she disagrees with me.

Rate your response. Choose from 1 (never) to 6 (always):

1 - 2 - 3 - 4 - 5 - 6

6. I explain the reasons behind my expectations.

Rate your response. Choose from 1 (never) to 6 (always):

1 - 2 - 3 - 4 - 5 - 6

7. I provide comfort and understanding when my child is upset.

Rate your response. Choose from 1 (never) to 6 (always):

1 - 2 - 3 - 4 - 5 - 6

8. I compliment my child.

Rate your response. Choose from 1 (never) to 6 (always):

1 - 2 - 3 - 4 - 5 - 6

9. I consider my child’s preferences when I make plans for the family (e.g., weekends away and holidays).

Rate your response. Choose from 1 (never) to 6 (always):

1 - 2 - 3 - 4 - 5 - 6

10. I respect my child’s opinion and encourage him/her to express them.

Rate your response. Choose from 1 (never) to 6 (always):

1 - 2 - 3 - 4 - 5 - 6

11. I treat my child as an equal member of the family.

Rate your response. Choose from 1 (never) to 6 (always):

1 - 2 - 3 - 4 - 5 - 6

12. I provide my child with reasons for the expectations I have for him/her.

Rate your response. Choose from 1 (never) to 6 (always):

1 - 2 - 3 - 4 - 5 - 6

13. I have warm and intimate times together with my child.

Rate your response. Choose from 1 (never) to 6 (always):

1 - 2 - 3 - 4 - 5 - 6

Authoritarian parenting style:

1. When my child asks me why he/she has to do something, I tell him/her it is because I said so, I am your parent, or because that is what I want.

Rate your response. Choose from 1 (never) to 6 (always):

1 - 2 - 3 - 4 - 5 - 6

2. I punish my child by taking privileges away from him/her (e.g., TV, games, and visiting friends).

Rate your response. Choose from 1 (never) to 6 (always):

1 - 2 - 3 - 4 - 5 - 6

3. I yell when I disapprove of my child’s behavior.

Rate your response. Choose from 1 (never) to 6 (always):

1 - 2 - 3 - 4 - 5 - 6

4. I explode in anger toward my child.

Rate your response. Choose from 1 (never) to 6 (always):

1 - 2 - 3 - 4 - 5 - 6

5. I spank my child when I don’t like what he/she does or says.

Rate your response. Choose from 1 (never) to 6 (always):

1 - 2 - 3 - 4 - 5 - 6

6. I use criticism to make my child improve his/her behavior.

Rate your response. Choose from 1 (never) to 6 (always):

1 - 2 - 3 - 4 - 5 - 6

7. I use threats as a form of punishment with little or no justification.

Rate your response. Choose from 1 (never) to 6 (always):

1 - 2 - 3 - 4 - 5 - 6

8. I punish my child by withholding emotional expressions (e.g., kisses and cuddles).

Rate your response. Choose from 1 (never) to 6 (always):

1 - 2 - 3 - 4 - 5 - 6

9. I openly criticize my child when his/her behavior does not meet my expectations.

Rate your response. Choose from 1 (never) to 6 (always):

1 - 2 - 3 - 4 - 5 - 6

10. I find myself struggling to try to change how my child thinks or feels about things.

Rate your response. Choose from 1 (never) to 6 (always):

1 - 2 - 3 - 4 - 5 - 6

11. I feel the need to point out my child’s past behavioral problems to make sure he/she will not do them again.

Rate your response. Choose from 1 (never) to 6 (always):

1 - 2 - 3 - 4 - 5 - 6

12. I remind my child that I am his/her parent.

Rate your response. Choose from 1 (never) to 6 (always):

1 - 2 - 3 - 4 - 5 - 6

13. I remind my child of all the things I am doing and I have done for him/her.

Rate your response. Choose from 1 (never) to 6 (always):

1 - 2 - 3 - 4 - 5 - 6

Permissive parenting style:

1. I find it difficult to discipline my child.

Rate your response. Choose from 1 (never) to 6 (always):

1 - 2 - 3 - 4 - 5 - 6

2. I give in to my child when he/she causes a commotion about something.

Rate your response. Choose from 1 (never) to 6 (always):

1 - 2 - 3 - 4 - 5 - 6

3. I spoil my child.

Rate your response. Choose from 1 (never) to 6 (always):

1 - 2 - 3 - 4 - 5 - 6

4. I ignore my child’s bad behavior.

Rate your response. Choose from 1 (never) to 6 (always):

1 - 2 - 3 - 4 - 5 - 6
